# Reproducibility and feasibility of optic nerve diffusion MRI techniques: single-shot echo-planar imaging (EPI), readout-segmented EPI, and reduced field-of-view diffusion-weighted imaging

**DOI:** 10.1186/s12880-022-00814-5

**Published:** 2022-05-24

**Authors:** Fanglu Zhou, Qing Li, Xiaohui Zhang, Hongli Ma, Ge Zhang, Silin Du, Lijun Zhang, Thomas Benkert, Zhiwei Zhang

**Affiliations:** 1 Department of Radiology, The First Affiliated Hospital of Chongqing Medical University, No. 1, Youyi Road, Yuzhong District, Chongqing, China; 2 MR Collaborations, Siemens Healthcare Ltd., Shanghai, China; 3 MR Application Predevelopment, Siemens Healthcare GmbH, Erlangen, Germany

**Keywords:** Optic nerves, Diffusion-weighted imaging, Zoomed EPI, RESOLVE

## Abstract

**Background:**

Diffusion-weighted imaging (DWI) is an essential technique for optic nerve diseases. However, the image quality of optic nerve DWI is decreased by the distortions and artifacts associated with conventional techniques. In order to establish this method as a critical tool in optic nerve diseases, reproducibility and feasibility of new technical and conventional approaches of DWI need to be systematically investigated.

**Methods:**

DWIs were acquired using ss-EPI, readout-segmented EPI (rs-EPI) DWI, and reduced field-of-view (rFOV) DWI. 26 volunteers (mean age 31.2 years) underwent repeated MRI examinations in order to assess scan–rescan reproducibility and accuracy. The apparent diffusion coefficient (ADC) values (three ROIs were measured on each side) were determined to evaluate the reproducibility of each sequence and the differences between the three techniques. To quantify the geometric distortion artifacts, the length of optic nerve and the maximum angle of optic nerve were defined and compared to T2-weighted imaging. In addition, two readers evaluated four different aspects of image quality on 5-point Likert scales.

**Results:**

rs-EPI DWI (ICCs: 0.916, 0.797 and 0.781) and rFOV DWI (ICCs: 0.850, 0.595 and 0.750) showed higher reproducibility (ICCs: ROI_1_, ROI_2_ and ROI_3_) of mean ADC value in all three ROIs than ss-EPI DWI (ICCs: 0.810, 0.442 and 0.379). The quantitative analysis of geometric distortion yielded a higher agreement of both rs-EPI DWI and rFOV DWI with T2-weighted imaging than ss-EPI. rs-EPI DWI (2.38 ± 0.90) and rFOV DWI (2.46 ± 0.58) were superior to ss-EPI DWI (1.58 ± 0.64) with respect to overall image quality and other aspects of image quality, each with *P* < 0.05. The mean ADC values of rFOV DWI were significantly lower than those of rs-EPI DWI and ss-EPI DWI in all three ROIs (*P* < 0.001).

**Conclusions:**

Both rs-EPI DWI and rFOV-EPI DWI are suitable techniques for the assessment of diffusion restriction and provide significantly improved image quality compared with ss-EPI DWI. For methods using the same acquisition time, rFOV DWI is superior to ss-EPI DWI, while rs-EPI showed an overall superiority, although this technique took 47% longer to perform.

## Background

Optic nerve diseases cause severe visual disturbances that, currently, cannot be diagnostically confirmed on ocular examination [1]. Orbital magnetic resonance imaging (MRI) is a widely utilized method to analyze various optic diseases such as optical neuritis. The wide application of orbital MRI in clinical also highly valued functional MR technology, particularly diffusion-weighted imaging (DWI) [2–5]. Single-shot echo-planar imaging (ss-EPI) is a conventional DW technique for rapid assessment of diffusion restriction and was commonly used in clinical evaluation and differential diagnosis of optic neuritis [6, 7]. But in clinical circumstances, ss-EPI DWI of optic nerve suffers from partial-volume effects, magnetic susceptibility artifacts, chemical shift artifacts, and low image quality on account of the small dimension of the nerves and low resolution [8]. Recent studies have indicated that reduced field-of-view (rFOV) DWI provides outstanding improvements in image quality of the spinal cord [9, 10], pancreas [11], prostate [12, 13], breast [14], thyroid nodules [15], and optic nerve [16]. Literature has demonstrated that rFOV DWI showed an improvement in subjective image quality for optic nerve in the intraorbital segment compared with ss-EPI [17, 18]. Moreover, Readout-segmented echo-planar imaging (rs-EPI) is a promising technique that has already been reported to increase image quality in orbital imaging [19]. Therefore, the present study focused on comparing the reproducibility and feasibility of current state-of-the-art diffusion techniques for their application in optic nerve analyses.

## Methods

### Subjects

From December 2019 to April 2020, 33 healthy volunteers were recruited to undergo MRI scans of the optic nerve. All volunteers received and signed informed consent before undergoing routine MRI and DWI examination of the orbit. All subjects were aged 18 years or older and had no history of neurological disorders, amblyopia, or optic nerve diseases. Exclusion criteria included the following: (1) subjects with contraindication to MRI examination (such as pacemaker installation, metal implants in the body, claustrophobia, etc.); (2) the images cannot be observed due to noticeable motion artifacts, susceptibility artifacts, etc.

### MRI protocol

MRI was performed with a 3T MR scanner (MAGNETOM Skyra, Siemens Healthcare, Erlangen, Germany) using a 20-channel head matrix coil, and gradients with a peak amplitude of 45 mT/m and slew rate of 200 T/m/s. Imaging sequences included T2w imaging, and DWIs were acquired using ss-EPI, high-resolution rs-EPI (readout segmentation of long variable echo trains, RESOLVE™, Siemens Healthcare), and a prototype rFOV-EPI (syngo ZOOMit™, Siemens Healthcare). The imaging protocol was repeated on each of the subjects at an interval of 20–40 min. Volunteers were placed in a comfortable supine position, and a sponge was positioned to limit head movement.

B0 inhomogeneity can result in a variety of artifacts, including geometric distortion, image blurring, especially in EPI sequences [20]. For a fair comparison, the standard B0 shimming was used in all the sequences; The upside of utilizing modeled shim fields is that there are no issues with different FOVs or different resolutions [20]. The bandwidth was adjusted to the optimal value to make the echo spacing close to the minimum value to reduce image distortion in all three DWI sequences. Additionally, GRAPPA (GeneRalized Autocalibrating Partially Parallel Acquisitions) was applied in each of the three DWI sequences to reduce susceptibility changes at tissue interfaces [21].

The rFOV DWI sequence used the ‘Excitation Model’ of ZOOMit, replacing the traditional pulse with slice selective excitation. Different from conventional zoomed technology, the advanced non-parallel transmission (non-PTX) zoomed-DWI was performed with a rotation of the field of excitation (8 deg.) to reduce distortion and echo time and correspondingly improve image quality despite B0 inhomogeneities [22]. In addition, averaging (10 averages) was applied to improve the evaluation accuracy of the ADC values. Moreover, the advanced non-PTX might be more applicable in clinical practice because there is no need for parallel transmission configuration [22]. The majority of imaging parameters of rFOV EPI were referred to conventional ss-EPI DWI, and the FOV was reduced to 120 × 98 mm^2^, which covered the whole of the orbits region and the optic chiasma. The inplane resolution was 1.3 × 1.3 mm^2^, resulting in the scan time was almost the same as the ss-EPI DWI. To achieve inplane resolution was almost the same in rs-EPI DWI (1.1 × 1.1 mm^2^) and rFOV EPI, readout partial Fourier of 5/8 and readout segments of 7 were applied in rs-EPI DWI, but with the longest scan time of 3:12 min. The specific parameters of three DWI sequences are listed in Table 1.

**Table 1:** Magnetic resonance imaging sequence parameters

	T2w imaging	rs-EPI DWI	rFOV DWI	ss-EPI DWI
TR (ms)	4400	4650	3500	3700
TE (ms)	99	68	65	80
FOV (mm^2^)	180 × 180	180 × 108	120 × 98	210 × 210
Voxel size (mm^2^)	0.5 × 0.5 × 2.5	1.1 × 1.1 × 2.5	1.3 × 1.3 × 2.5	1.8 × 1.8 × 2.5
b-value (s/mm^2^)	–	0, 1000	0, 1000	0, 1000
Diffusion directions	**–**	3	3	3
Averages per b-value	–	2,2	2,10	2,10
PAT mode	GRAPPA	GRAPPA	GRAPPA	GRAPPA
Acceleration factor PE	2	2	2	2
In-plane resolution (mm^2^)	0.5 × 0.5	1.1 × 1.1	1.3 × 1.3	1.8 × 1.8
Slice thickness (mm)	2.5	2.5	2.5	2.5
Base resolution	384	160	96	120
Echo spacing (mm)	11.0	0.36	0.99	0.76
Bandwidth (Hz/px)	250	679	1158	1488
EPI factor	–	96	78	120
B0 Shim mode	Standard	Standard	Standard	Standard
TA (min)	1:47	3:12	2:03	2:11

rs-EPI DWI, readout-segmented echo-planar imaging diffusion-weighted imaging; rFOV, reduced field-of-view; ss-EPI, single-shot echo-planar imaging; TE, echo time; TR, repetition time, TA, acquisition time, GRAPPA: generalized autocalibrating partially parallel acquisitions

### Evaluation and analysis

#### ADC values

**Fig. 1:**
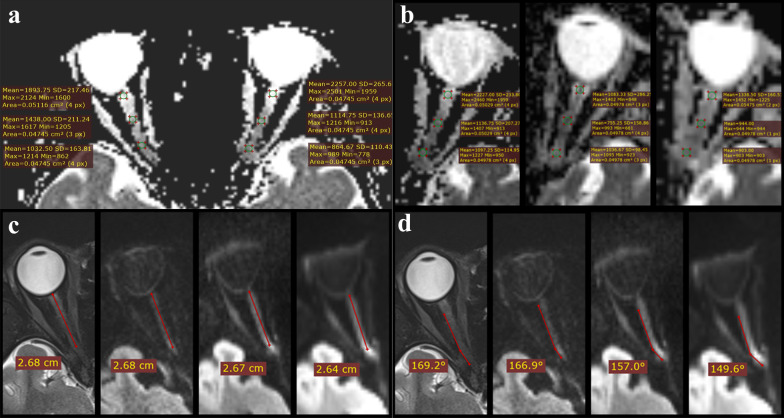
Examples of quantitative optic nerve measurements. **a** Measurement of ADC values. Three ROIs were measured on each side. **b**, Measurement of ADC values. Three ROIs were measured on the left side, along with (left to right) images of ADC map of rs-EPI DWI, ADC map of rFOV-EPI DWI and ADC map of ss-EPI DWI, respectively; **c** the length of the intraorbital segment of the optic nerve is shown, along with (left to right) images of T2w, rs-EPI DWI at b value = 1000 s/mm^2^, rFOV-EPI DWI at b value = 1000 s/mm^2^ and ss-EPI DWI at b value = 1000 s/mm^2^ (respectively); **d** Maximum angles of the optic nerve are shown, along with (left to right) images of T2w, rs-EPI DWI at b value = 1000 s/mm^2^, rFOV-EPI DWI at b value = 1000 s/mm^2^ and ss-EPI DWI at b value = 1000 s/mm^2^ (respectively). Compared with the T2w images, the DWI images had different degrees of distortion, and the angle of the optic nerve became smaller. ADC, apparent diffusion coefficient; rs-EPI DWI, readout-segmented echo-planar imaging diffusion-weighted imaging; rFOV, reduced field-of-view; ss-EPI, single-shot echo-planar imaging

The ADC values of bilateral optic nerves were assessed on the ADC maps at slice that covered the largest extent of the optic nerves from ss-EPI DWI, rFOV DWI, and rs-EPI DWI images. The measurements were made in each of the two repeated scans to evaluate the reproducibility. Three regions of interest (ROIs) using the shape of a circle or ellipse and encompassing an area of 50 (± 3) mm^2^ were measured on each side, as shown in Fig. 1. ROI_1_s were performed on the starting point of the intraorbital segment of the optic nerve and were centered within 3 mm of the back of the eyeball. ROI_3_s were performed on the end of the intraorbital segment of the optic nerve. The center of ROI_2_s was at the midpoint of the line between the center of ROI_1_s and the center of ROI_3_s. ROI was drawn on the image of the first scan and sent to that of the second scan for the same sequence. Each ROI was measured three times, and the means were recorded as a result.

#### Distortion artifacts analysis

The length of the intraorbital segment of the optic nerve and the maximum angle at which the optic nerve bends were defined to quantify the geometric distortion. The length and the maximum angle of the intraorbital segment of the optic nerve were measured on each side of the eye on this slice which covered the largest extent of the optic nerve of the EPI sequences with a b-value of 1000 s/mm^2^; T2w imaging was used as a reference. The quantitative analysis was performed using RadiAnt Dicom Viewer (version 5.5.0). An example is shown in Fig. 1.

#### Image quality

We compared the image quality of rs-EPI DWI, rFOV DWI, and ss-EPI DWI using a 5-point scale visual evaluation. Two radiological technologists with 4 and 7 years of experience independently evaluated image quality in the qualitative assessments of the four aspects on the *b* = 1000 s/mm^2^ images; The FOV of the DWIs was adapted so that the readers were blinded to the used sequence. The detailed evaluation criteria of image quality are shown in Table 2. Mean scores were calculated from those of the two readers. Similarly, the signal-to-noise ratio of images was measured. However, due to the distortion of parallel acquisition sequence, as well as the lack of inclusion of background air in the rFOV DWI images, consistent with a previous report regarding estimating SNR in the presence of parallel imaging, the estimated signal-to-noise ratio (eSNR, the ratio between the mean and standard deviation (SD) of the given ROI.) corresponding to each ROI on the b-1000 images was calculated [23]. The ROIs were placed on an area showing visually normal signal intensity and an absence of artifacts (The ROIs of the three sequences were placed in the grey matter region of the left frontal lobe at the same slice) and had a mean size of 50 (± 3) mm^2^. The mean and SD of all ROIs were recorded.

**Table 2:** Evaluation criteria of image quality

	Overall image quality	Artifacts (degree of susceptibility)	Image blurring	Distortion (compared with the corresponding T2w imaging)
0	Non-diagnostic	No artifacts	None	None
1	Poor	Minor artifacts	Minimal	Minimal
2	Fair	Moderate artifacts	Moderate	Moderate
3	Good	Severe artifacts	Considerable	Considerable
4	Excellent	Very severe artifacts	Pronounced	Pronounced

### Statistical analysis

The statistical data were analyzed using MedCalc 11.5.0 (https://www.medcalc.org/) and SPSS version 22.0 (Chicago, IL, USA). A *P* value of less than 0.05 was considered statistically significant.

Shapiro–Wilk was used to check the normal distribution of the data. The reproducibility of the mean ADC of two repeated MR examinations was established using 95% Bland–Altman limits of agreements (BA-LA) and intraclass correlation coefficients (ICCs, two-way random). ANOVA or Friedman test (depending on whether fulfilling the assumption of the parametric test) was performed to compare the mean ADC values and eSNR of three sequences. In addition, a two-tailed pair Student’s t-test was used to compare the difference of mean ADC values between each pair of left and right nerves, respectively.

The agreement of the length of the intraorbital segment of the optic nerve and the maximum angle at which the optic nerve bends of the three techniques with T2w imaging was determined by the calculation of ICCs (two-way random). In the qualitative assessments of the five aspects of image quality, the mean scores of the two readers were calculated. Results are presented as mean ± SD. Bonferroni correction for multiple comparisons was performed after the overall significance was achieved. All aspects of image quality were evaluated using the Wilcoxon signed-rank test.

## Results

Twenty-six volunteers (6 men, 20 women; mean age, 31.2 years; range, 22–55 years) of the total 33 were performed the following parameter and image quality evaluations (seven volunteers were excluded, four volunteers dropped out of the experiment due to personal reasons and three were due to severe motion artifacts and susceptibility artifacts of images).

### Reproducibility

**Fig. 2:**
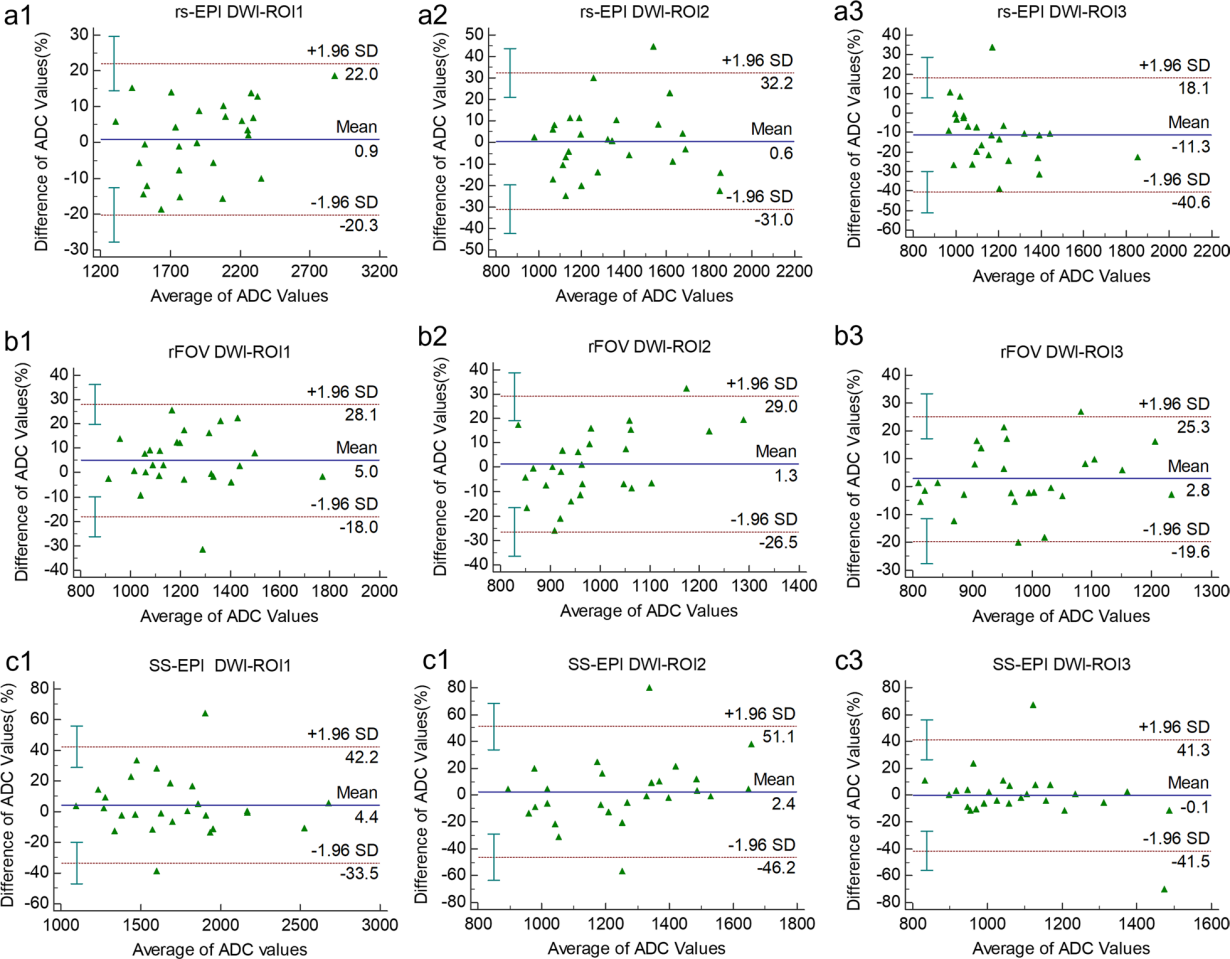
Reproducibility and accuracy of ADC values assessment. Bland–Altman plots show reproducibility of mean ADC values in the optic nerve during repeat exams. rs-EPI DWI, readout-segmented echo-planar imaging diffusion-weighted imaging; rFOV, reduced field-of-view; ss-EPI, single-shot echo-planar imaging. **a1**–**a3** Reproducibility evaluation of mean ADC values of rs-EPI DWI. **a1**–**a3** are the Bland–Altman plots of ROI_1_, ROI_2_, and ROI_3_, respectively. **b1–b3** Reproducibility evaluation of mean ADC values of rFOV DWI. **b1–b3** are the Bland–Altman plots of ROI_1_, ROI_2_, and ROI_3_, respectively. **c1–c3** Reproducibility evaluation of mean ADC values of ss-EPI DWI. **c1–c3** are the Bland–Altman plots of ROI_1_, ROI_2_, and ROI_3_, respectively. In each plot, the solid line represents the average value (percentage) of the difference between the two scans; the dashed lines represent the 95% CI (confidence intervals) of the difference; the blue error bars close to the y-axis represent 95% CI of upper limit, 95% CI of arithmetic mean, 95% CI of the lower limit, respectively

The results from the 95% Bland–Altman limits of agreements (BA-LA) and intraclass correlation coefficients (ICCs, two-way random) are shown in Table 3. Bland–Altman plots are also given to show reproducibility of mean ADC values in the optic nerve during repeat MRI exams (Fig. 2). rs-EPI DWI showed the highest reproducibility of mean ADC value in three ROIs (ICCs > 0.75), especially ROI_1_ (ICCs = 0.916). In comparison, ss-EPI DWI showed the lowest reproducibility, especially in ROI_2_ and ROI_3_. For rs-EPI DWI, the mean ADC value of the middle segment of the optic nerve was relatively unstable, and the reproducibility was the lowest. In contrast, the reproducibility of the mean ADC value of ROI_1_ was significantly higher than that of ROI_2_ and ROI_3_ for all three DWI sequences. In addition, the reproducibility of the mean ADC value of ROI_2_ and ROI3 of rs-EPI and ROI_3_ of rFOV-DWI were good (ICCs > 0.75).

**Table 3:** Reproducibility of ADC Values in repeated MR exams

	rs-EPI	rFOV-DWI	ss-EPI
ROI_1_			
ICCs	0.916	0.850	0.810
BA-LA	− 20 to 22	− 18 to 28.1	− 33.5 to 22.2
ROI_2_			
ICCs	0.797	0.595	0.442^a^
BA-LA	− 31 to 32.2	− 26.5 to 29	− 46.2 to 51.1
ROI_3_			
ICCs	0.781	0.750	0.379^a^
BA-LA	− 40.6 to 18.1	− 19.6 to 25.3	− 41.5 to 41.3

BA-LA (in %), the 95% Bland–Altman limits of agreements; ICCs, intraclass correlation coefficients (two-way random); rs-EPI DWI, readout-segmented echo-planar imaging diffusion-weighted imaging; rFOV, reduced field-of-view; ss-EPI, single-shot echo-planar imaging; ROI, region of interest

^a^*P* > 0.05

### ADC values

There were no statistically significant differences in mean ADC values between the ROIs in the left and right optic nerve from the three techniques (*P* > 0.05). Therefore, the ADC values from the three methods were compared using the average ADC values of the left and right optic nerves of each ROI.

For each DWI technique, mean ADC values among the three different ROIs showed statistically significant differences (*P* < 0.01). In all three DWI sequences, the mean ADC value of ROI_1_ was the highest, while the mean ADC value of ROI_3_ was the lowest (Table 4). Multiple comparisons showed the following: a) there were significant differences between ROI_1_ and ROI_2_/ROI_3_ in all three DWI methods (*P* < 0.01), and b) there were no significant differences between ROI_2_ and ROI_3_ in rs-EPI DWI (*P* = 0.294), rFOV DWI (*P* = 0.770) and ss-EPI DWI (*P* = 0.162).

**Table 4:** Results of quantitative evaluations of mean ADC values (10 × ^−6^ mm^2^/s)

	ROI_1_	ROI_2_	ROI_3_	F/χ^2^	*P*
First^a^					
rs-EPI DWI	1939.79 ± 432.73^d^	1344.62 ± 278.79^d^	1043.69 ± 199.62^c^	43.00^e^	< 0.001
rFOV-DWI	1251.02 ± 209.81^d^	998.69 ± 168.511^d^	995.99 ± 139.73^d^	18.20^f^	< 0.001
ss-EPI DWI	1744.47 ± 413.35^d^	1271.74 ± 310.28^d^	1088.33 ± 169.14^d^	36.54^e^	< 0.001
F/χ^2^	27.77^e^	21.77^e^	9.80^e^		
*P*	< 0.001	< 0.001	0.008		
Second^b^					
rs-EPI DWI	1908.60 ± 337.60^d^	1335.59 ± 284.62^d^	1249.13 ± 256.82^d^	38.40^f^	< 0.001
rFOV-DWI	1117.77 ± 277.55^c^	977.81 ± 94.24^d^	965.94 ± 116.86^d^	19.36^e^	< 0.001
ss-EPI DWI	1675.44 ± 429.45^d^	1225.84 ± 221.47^d^	1034.00 ± 220.31^c^	23.66^e^	< 0.001
F/χ^2^	29.96^e^	31.69^e^	24.92^e^		
*P*	< 0.001	< 0.001	< 0.001		

ADC, apparent diffusion coefficient; rs-EPI DWI, readout-segmented echo-planar imaging diffusion-weighted imaging; rFOV, reduced field-of-view; ss-EP, single-shot echo-planar imaging; ROI, region of interest

^a^Mean ADC values of the first scan

^b^Mean ADC values of the second scan

^c^Parameter value was a non-normal distribution, expressed as a median ± quartile range

^d^Parameter value was a normal distribution, expressed as mean ± standard deviation

^e^χ^2^ value of Friedman’s test

^f^F value of ANOVA

For each ROI, mean ADC values among the three different DWI techniques showed statistically significant differences (*P* < 0.001). In all three ROIs, the mean ADC value of rs-EPI DWI was the highest, while the mean ADC value of rFOV DWI was the lowest (Table 4). Mean ADC values of ROI_1_ (*P* = 0.058), ROI_2_ (*P* = 0.315) and ROI_3_ (*P* = 0.679) between rs-EPI DWI and ss-EPI DWI did not differ significantly. The mean ADC values of rFOV DWI were significantly lower than those of rs-EPI DWI and ss-EPI DWI in all three ROIs.

### Image quality

**Fig. 3:**
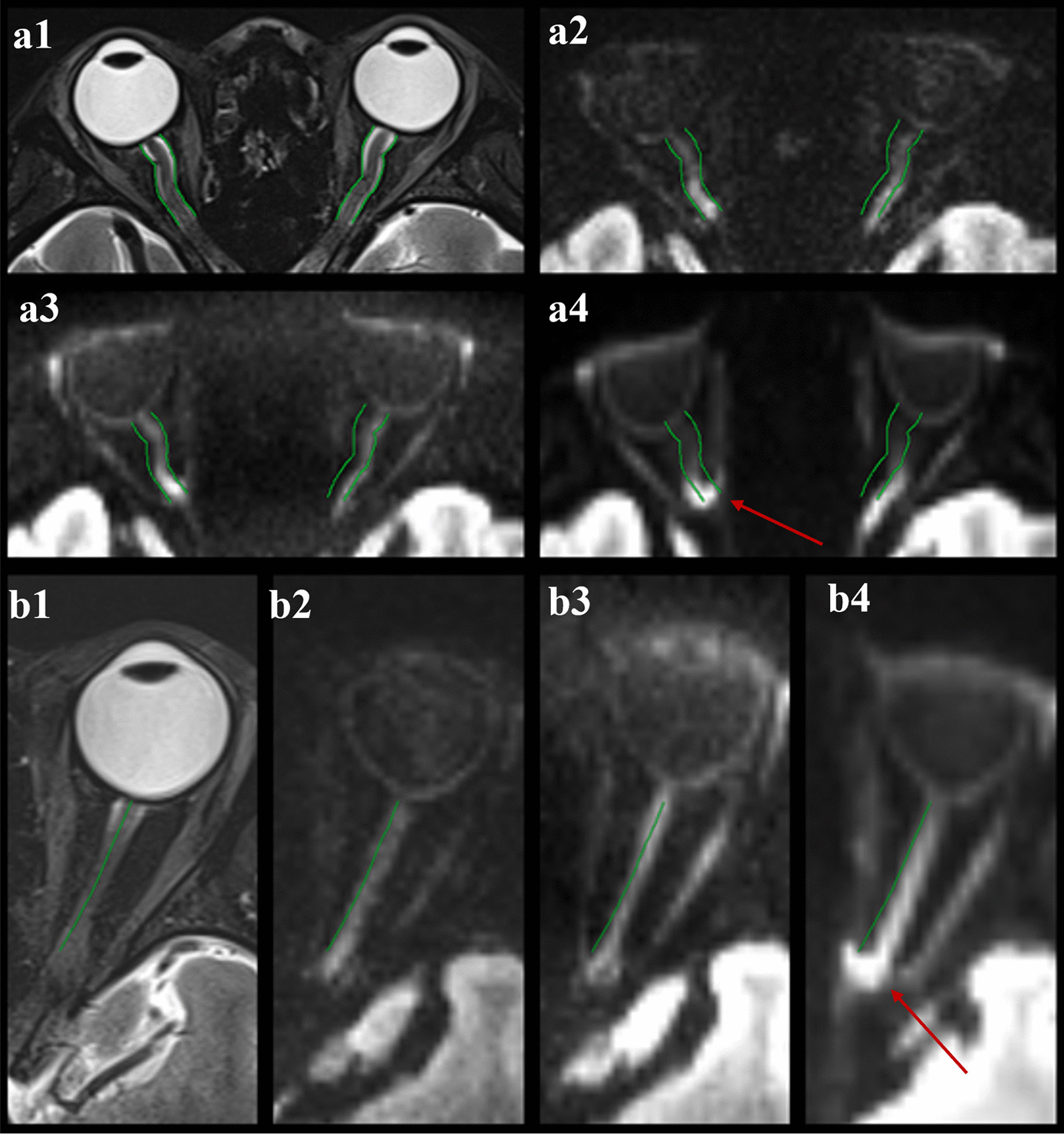
Examples of image quality and distortion of three DW techniques. **a** Imaging in a 28-year-old female volunteer. **a1–a4** images of T2w, rs-EPI DWI at b value = 1000 s/mm^2^, rFOV-EPI DWI at b value = 1000 s/mm^2^ and ss-EPI DWI at b value = 1000 s/mm^2^, respectively. **b** Imaging in a 25-year-old female volunteer. **b1**–**b4** images of T2w, rs-EPI DWI at b value = 1000 s/mm^2^, rFOV-EPI DWI at b value = 1000 s/mm^2^ and ss-EPI DWI at b value = 1000 s/mm^2^, respectively. Note the improved image quality and markedly reduced susceptibility distortions in the rs-EPI and rFOV-EPI sequences compared with ss-EPI. There are severe distortions due to the susceptibility artifacts caused by the air-bone-tissue interface on ss-EPI DWI (arrows in **a4**, **b4**). rs-EPI DWI, readout-segmented echo-planar imaging diffusion-weighted imaging; rFOV, reduced field-of-view; ss-EPI, single-shot echo-planar imaging

Using data pooled between the two technologists, the overall image quality of the DW images of rFOV DWI and rs-EPI DWI were significantly higher (*P* < 0.001) than the image quality of the ss-EPI DWI images (Fig. 3). Artifacts were significantly less severe (*P* < 0.001) in the rFOV DWI and rs-EPI DWI sequences compared with ss-EPI DWI. Likewise, distortion (*P* < 0.001) and image blurring (*P* < 0.001) of the DW images were rated significantly lower for the rFOV DWI and rs-EPI DWI sequence than ss-EPI DWI (Fig. 3). The results are shown in Table 5. Pairwise comparisons revealed significant differences between ss-EPI DWI and the other two sequences in image quality, artifacts, distortion, and image blurring, with *P* values of *P* = 0.001, *P* = 0.039, *P* < 0.001, and *P* < 0.001 (respectively) versus rs-EPI DWI; and *P* < 0.001, *P* = 0.012, *P* = 0.009, *P* < 0.001, and *P* < 0.001 versus rFOV DWI. For the comparison of rs-EPI DWI and rFOV DWI, there were significant differences in image blurring (*P* = 0.002) and distortion (*P* < 0.001), but the differences were not statistically significant for other image quality parameters. In terms of eSNR, there were statistically significant differences in eSNR among three DWI sequences. eSNR of rFOV DWI images (17.78 ± 4.16) was lower than that of ss-EPI DWI (26.77 ± 8.58), but was higher than that of rs-EPI DWI (10.18 ± 1.89), each with *P* < 0.01.

**Table 5:** Results of image quality evaluations

	rs-EPI DWI	rFOV DWI	ss-EPI DWI	*P*
Image quality	2.38 ± 0.90	2.46 ± 0.58	1.58 ± 0.64	< 0.001
Artifacts	1.96 ± 0.91	1.85 ± 0.67	2.27 ± 0.67	0.042
Distortion	1.5 ± 0.58	1.81 ± 0.63	3.04 ± 0.72	< 0.001
Image blurring	1.35 ± 0.48	1.73 ± 0.45	3.04 ± 0.53	< 0.001

rs-EPI DWI, readout-segmented echo-planar imaging diffusion-weighted imaging; rFOV, reduced field-of-view; ss-EPI, single-shot echo-planar imaging

### Distortion artifacts analysis

Results of distortion artifacts analysis are shown in Table 6. Regarding the anatomic agreement of the three EPI techniques assessed by ICCs, rs-EPI DWI and rFOV DWI showed stronger agreement (higher ICCs) than did ss-EPI DWI for both the length of the optic nerve and the maximum angle of the optic nerve. And the agreement of the maximum angle of the optic nerve was stronger than that of the length of the optic nerve in all DWI methods. The differences of the quantitative measurements in relation to T2w imaging (mean(range)) varied in the length of the left optic nerve (rs-EPI: 0.26 (0.01–0.87) mm, rFOV-EPI: 0.27(0–0.98) mm and ss-EPI :0.31 (0.01–0.97) mm), maximum angle of the left optic nerve (rs-EPI: 5.92(0–23.8)°, rFOV-EPI: 6.84 (1.2–20.7)° and ss-EPI: 11.7(0.2–58.4)°), length of the right optic nerve (rs-EPI: 0.21(0.03–0.56) mm, rFOV-EPI: 0.25(0.02–0.81) mm and ss-EPI: 0.34(0.02–0.92) mm), and maximum angle of the right optic nerve (rs-EPI: 7.8(0.7–20.6)°, rFOV-EPI: 8.88(0.4–25.8)° and ss-EPI: 11.85(0.3–53.5)°). The results of the difference were consistent with the results of ICCs. The differences between the length of the optic nerve and the angle and optic nerve of rs-EPI DWI and the T2WI image were the smallest, while geometric distortion was more obvious on the ss-EPI DWI images. Compared with the rs-EPI and rFOV-EPI, the length of the right optic nerve of ss-EPI DWI images was significantly shorter, and the angle of inward bending the middle and posterior optic nerve was larger (Fig. 3).

**Table 6:** Results of quantitative evaluations of anatomic measurements

	L-length (mm)	R-length (mm)	L-angle (°)	R-angle (°)
T2WI	2.44 ± 0.24	2.36 ± 0.18	169.61 ± 7.51	170.58 ± 7.12
rs-EPI DWI	2.25 ± 0.23	2.20 ± 0.23	165.24 ± 9.26	166.78 ± 11.02
ICCs^a^	0.691	0.734	0.993	0.989
95%CI^b^	0.341–0.855	0.426–0.877	0.986–0.997	0.977–0.995
rFOV DWI	2.21 ± 0.308	2.13 ± 0.28	165.64 ± 8.63	165.82 ± 11.24
ICCs^a^	0.687	0.667	0.992	0.985
95%CI^b^	0.334–0.853	0.281–0.846	0.984–0.996	0.969–0.993
ss-EPI DWI	2.17 ± 0.32	2.04 ± 0.31	158.92 ± 14.9	161.79 ± 17.72
ICCs^a^	0.659	0.675	0.978	0.971
95%CI^b^	0.275–0.840	0.297–0.850	0.954–0.990	0.939–0.987

rs-EPI DWI, readout-segmented echo-planar imaging diffusion-weighted imaging; rFOV, reduced field-of-view; ss-EPI, single-shot echo-planar imaging; L-length, the length of the left optic nerve; R-length, the length of the right optic nerve; L-angle, the maximum angle of the left optic nerve; R-angle, the maximum angle of the right optic nerve

^a^Anatomic agreement of three sequences with T2w imaging, measured by intraclass correlation coefficients (ICCs)

^b^95% confidence interval (CI) of ICCs. T2WI, T2 weighted imaging

## Discussion

Since DWI has been proven to be a critical functional technology in detecting and locating optic nerve disease lesions, for example, the use of DWI and the calculation of ADC values for evaluating optic neuritis (ON) has been reported [24, 25]. Using only DWI has high sensitivity and specificity in distinguishing acute from chronic ON. Differences in ADC values can reflect different pathogenesis of ON [26], and ADC values give a measure of axonal disruption in the chronic optic nerve lesion [27, 28]. Moreover, DWI has been used for the assessment of various diseases, such as trauma [29] and ischemia [30, 31], that involve the optic nerves. DWI can also be considered the first technique capable of identifying posterior ischemic optic neuropathy (PION) by identifying acute ischemic lesions of the optic nerve [32]. And ADC may serve as a useful tool for prognostication for Optic pathway glioma (OPG), a significantly higher mean ADC was seen in OPG that required therapy for tumor progression [33].

But DWI of the optic nerve is difficult in clinical circumstances, on account of the small dimension of the nerves, uncontrolled eye movements, and the high signal from cerebrospinal fluid or neighbouring fat within the orbital region [8]. ss-EPI DWI suffers from magnetic susceptibility artifacts, chemical shift artifacts, and low image quality. The present study found that rFOV DWI exhibited superior performance compared with ss-EPI DWI in all evaluated aspects, including blurring effects, image distortion, artifacts, lesion conspicuity, and image quality. Barker et al. also found that rFOV DWI provided improved subjective image quality of optic neuritis compared with ss-EPI DWI [34]. Owing to the readout-segmented k-space acquisition strategy, rs-EPI effectively reduces the image distortion caused by the large susceptibility variations and the T2* blur effect [35]. Therefore, the application of ss-EPI in optic nerve imaging has been limited in the past. In this study, we evaluated new technical approaches of DWI of the optic nerve.

rs-EPI DWI showed higher reproducibility than did ss-EPI DWI and rFOV DWI. In the present study, rs-EPI DWI appeared to be the most robust and reliable method. The rs-EPI arrangement partitions the k-space trajectory into numerous portions in the readout direction. Accordingly, TE and encoding times can be decreased, and movement correction can be performed utilizing a 2D navigator correcting motion-induced, non-linear phase errors [36, 37]. However, the problem is that the acquisition time is longer on account of multiple TR intervals. Seeger et al. found that rFOV-EPI (2:45 s) showed improved image quality, the most accurate tumor delineation, and the best differentiation from retinal detachments compared with ss-EPI (2:14 s) and rs-EPI (3:07 s) in patients with uveal melanomas [38]. In the present study, the reproducibility of the mean ADC value of rFOV DWI (2:03 s) was better than that of ss-EPI DWI (2:11 s), but the reproducibility of ROI_2_ was lower than for rs-EPI DWI (3:12 s). The mean ADC value of rFOV DWI was significantly lower than those of rs-EPI DWI and ss-EPI DWI, which is consistent with the results of Seeger et al. [18]. We speculate the reason for these findings may be that we applied FOV rotation to remove the potential folding artifacts of rFOV-DWI and used complex averages to improve the ADC estimation. Furth more, rFOV DWI overcomes the major problem of low specific absorption rate and low spatial resolution facing DWI on the optic nerve as the reproducibility of the ADC values of the ss-EPI DWI sequence is relatively low. It is not recommended to use the ADC values of the ss-EPI DWI sequence as an indicator in follow-up cases.

Common diseases of the optic nerve include optic neuritis, ischemic optic neuropathy, optic nerve tumors, etc. The clinical manifestations and involvement of optic nerve segments in different diseases are different. For example, inflammation of the optic nerve can be involved unilaterally or bilaterally, and it can also be involved in long segments. In the application of the ADC values of the optic nerve, the ADC value of the contralateral side of the diseased optic nerve is usually used as the control region. Still, the ADC value of the area with no obvious abnormal signal is usually measured in time for bilateral involvement as a reference. ADC measurement of the optic nerve is challenging due to the small diameter of the optic nerve because there is the potential of partial volume averaging with surrounding CSF, fat, and osseous structures. Susceptibility artifacts caused by the air-bone-tissue interface also affected the ROI placement over the optic nerve. Therefore, three ROIs in different positions were placed to explore the possible differences in ADC values. The comparison of ADC values shows that the ADC value of ROI_1_ has the best reproducibility and is significantly higher than the ADC values of ROI_2_ and ROI_3_, indicating that the signal of the optic nerve behind the global is continuously affected by partial volume averaging with the high signal of the eyeball and needs to be taken into consideration to rule out false positives. And when choosing the control region for comparison, the area close to the eyeball should be avoided. There are both good reproducibility in ADC values of ROI_2_ and ROI_3_, and the difference between ADC of ROI_2_ and ROI_3_ is not statistically significant, which may indicate that compared to ROI_2_, ROI_3_ does not suffer more inaccurate due to susceptibility artifacts caused by the air-bone-tissue interface and partial volume averaging with the surrounding air and osseous structures.

Distortion creates a challenge for diffusion MRI of the optic nerve, especially at high field strengths. The optic nerve is surrounded by fat, muscle, bone, and air leading to large susceptibility changes that distort diffusion-weighted EPI images. Our research found that rs-EPI DWI showed the highest agreement with T2w imaging in terms of the length and angle of the optic nerve compared with those obtained with rFOV DWI and ss-EPI. The distortion in the length of the optic nerve may indicate the degree of compression of the image in the long axis of the optic nerve. The distortion of the angle of the optic nerve may represent the distortion ratio of the x-axis compared with the y-axis. Previous studies have reported quantitative evaluations of the degree of distortion in this process. In the study of Thierfelder et al. [13], the rFOV DWI of the prostate showed a stronger correlation with the T2w images in the coronal and sagittal diameters as well as in the prostate volume, compared with those obtained by ss-EPI DWI, yielding ICCs of 0.948 for the coronal diameter, 0.858 for the sagittal diameter, and 0.938 for the prostate volume. These observations are consistent (to a certain extent) with the results of the present study. Specifically, we observed that rFOV DWI has a good correlation with T2w images in terms of angle. Still, there is distortion in the long axis of the optic nerve, which is like the fact that the ICC coefficient of the prostate also is low in the longitudinal axis direction. We speculate that the lower ICC coefficient in the longitudinal axis may be due to the susceptibility artifacts caused by the air-bone-tissue interface.

### Limitations

There are several limitations in this study. Firstly, our quantitative analysis was restricted to a global comparison of the optic nerve length and angle. It might be of interest to determine how well other parameters in rFOV DWI or rs-EPI DWI correlate with T2w imaging. Additionally, the present investigation only evaluated the intraorbital segment of the optic nerve and did not examine other segments of this nerve. We have made the comparison between two product DWI sequences and a prototype ZOOMit DWI sequence; there are still some other techniques to reduce distortion and improve image quality that we haven’t compared, like BLADE DWI and ss-EPI with two b0 acquisitions of opposite phase-encoding directions, etc. Secondly, the methods tested in this study are not yet widely used in clinical practice. So, it seems that they may not be available on the specific vendor's platform. Furthermore, the performance results of the three sequences and the results of the image quality assessment may vary depending on the specific hardware. Therefore, the findings reported in this study are specific to the hardware of these tests. Thirdly, the number of patients examined was only adequate for an exploratory study; additional work with a larger population and patients with pathology is warranted to corroborate our findings. Although ADC values of different methods are compared in volunteers, there is no reference to the gold standard, so ADC phantom can be further used to verify the standard in future experiments.

## Conclusion

rs-EPI DWI and rFOV-EPI DWI enable high-quality imaging in the intraorbital segment of the optic nerve. Both rs-EPI DWI and rFOV-EPI DWI are suitable techniques for the assessment of diffusion restriction and provide significantly improved image quality compared with ss-EPI DWI. For methods using the same acquisition time, rFOV DWI is superior to ss-EPI DWI, while rs-EPI is the best choice, although this technique took 47% longer to perform.

## Data Availability

The datasets generated and/or analysed during the current study are not publicly available due to the confidentiality statement of informed consent (In informed consent: “Information that could identify you will not be disclosed to members outside the research team unless you have given your permission. All study members and study sponsors are required to keep your identity confidential”). But are available from the corresponding author on reasonable request.

## Abbreviations

ADC: Apparent diffusion coefficient
DWI: Diffusion-weighted imaging
EPI: Echo-planar imaging
rs-EPI DWI: Readout-segmented echo-planar imaging diffusion-weighted imaging
rFOV: Reduced field-of-view
ss-EPI: Single-shot echo-planar imaging
FOV: Field of view
ROI: Region of interest
ICC: Intraclass correlation coefficient
TE: Echo time
TR: Repetition time
TA: Acquisition time
GRAPPA: Generalized autocalibrating partially parallel acquisitions
BA-LA (in %): The 95% Bland–Altman limits of agreements
